# Multifactorial Analysis of Mortality in Screening Detected Lung Cancer

**DOI:** 10.1155/2018/1296246

**Published:** 2018-05-16

**Authors:** Subba R. Digumarthy, Ruben De Man, Rodrigo Canellas, Alexi Otrakji, Ge Wang, Mannudeep K. Kalra

**Affiliations:** ^1^Division of Thoracic Imaging, Department of Radiology, Massachusetts General Hospital, Boston, MA 02114, USA; ^2^Department of Biomedical Engineering, Rensselaer Polytechnic Institute, Troy, NY 12180, USA

## Abstract

We hypothesized that severity of coronary artery calcification (CAC), emphysema, muscle mass, and fat attenuation can help predict mortality in patients with lung cancer participating in the National Lung Screening Trial (NLST). Following regulatory approval from the Cancer Data Access System (CDAS), all patients diagnosed with lung cancer at the time of the screening study were identified. These subjects were classified into two groups: survivors and nonsurvivors at the conclusion of the NLST trial. These groups were matched based on their age, gender, body mass index (BMI), smoking history, lung cancer stage, and survival time. CAC, emphysema, muscle mass, and subcutaneous fat attenuation were quantified on baseline low-dose chest CT (LDCT) for all patients in both groups. Nonsurvivor group had significantly greater CAC, decreased muscle mass, and higher fat attenuation compared to the survivor group (*p* < 0.01). No significant difference in severity of emphysema was noted between the two groups (*p* > 0.1). We thus conclude that it is possible to create a quantitative prediction model for lung cancer mortality for subjects with lung cancer detected on screening low-dose CT (LDCT).

## 1. Introduction

Each year, nearly 1.6 million people worldwide die from lung cancer which accounts to almost a quarter of all cancer-related deaths [1]. Effective treatment and patient survival depend on the ability to detect lung cancer in the early stages [2]. Lung cancer screening is an effective method to improve survival in high risk groups, as it allows detection of early stage cancers. Efficacy of lung cancer screening with low-dose computed tomography (LDCT) has been validated in several large-scale clinical trials [3–8].

The Early Lung Cancer Action Program (ELCAP) compared conventional chest radiography with LDCT for lung cancer screening with more than 30,000 subjects [7]. The National Lung Screening Trial (NLST) compared LDCT with the chest radiography in more than 50,000 current or former smokers who met the various inclusion criteria [8]. LDCT was found to be effective in detecting more lung cancers in the early stages as compared to chest radiography. Although NLST demonstrated improved survival following early detection of lung cancer, there were nonsurvivors in the trial as well.

We hypothesized that patient survival in subjects with lung cancer detected on LDCT in the NLST cohort can be predicted based on risk factors that predict mortality in other nonscreening detected cancers [9–12]. These factors include skeleton muscle area, subcutaneous fat attenuation, CAC, and emphysema. The purpose of our study was to assess differences in skeletal muscle area, subcutaneous fat attenuation, coronary artery calcification (CAC), and emphysema in surviving and nonsurviving subjects with lung cancers detected in the NLST.

## 2. Methods

### 2.1. Approvals

All study data were obtained from the National Cancer Institute Cancer Data Access System (CDAS) maintained by the National Institute of Health (NIH) [13]. Access to study data was granted following a formal review and approval by the National Cancer Institute (NCI). All shared datasets in the CDAS are completely deidentified of all confidential patient identifiers, which were labeled with a unique 6-digit identification number. None of the coauthors have any pertinent financial disclosure. Since the study data did not belong to our institution, the institutional review board for human subjects deemed our study as exempt from need for approval.

### 2.2. Subjects

**Figure 1:**
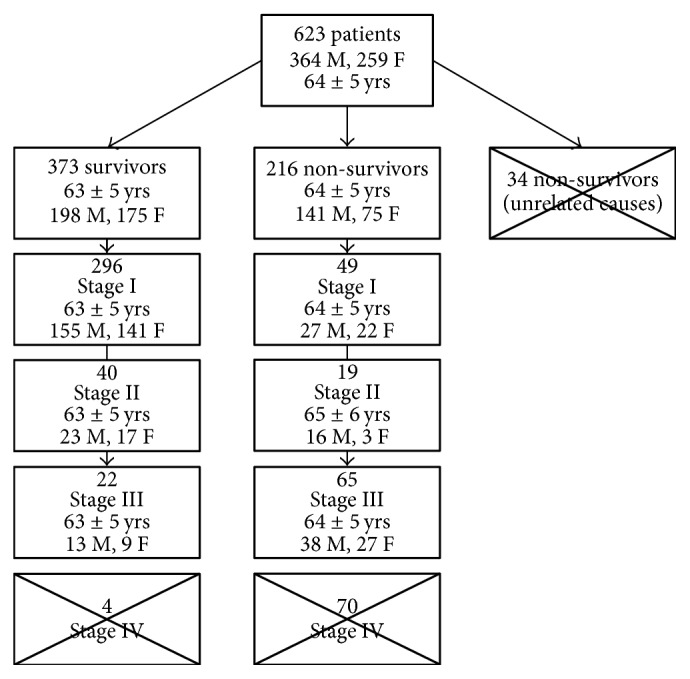
Method used to select patients for study.

From the shared NLST master sheet of all enrolled subjects, we identified 623 subjects (entire data of NLST are not shared) in whom lung cancer was detected on screening LDCT (column name: can scr). Data for these 623 subjects were then sorted based on their final status at conclusion of the NLST (column name: finaldeathLC). There were 373 survivors and 216 nonsurvivors. Of the 216 nonsurvivors, 34 subjects died of unrelated causes and were excluded. Patients (*n* = 182 subjects) were considered nonsurvivors if the death was related to lung cancer, its work-up (such as biopsy), or treatment. Next, we sorted the two groups based on the stage of lung cancer at the time of initial detection (nonsurvivors group: stage 1 (*n* = 49 subjects), stage II (*n* = 19), stage III (*n* = 65), and stage IV (*n* = 70); survivor group: stage 1 (*n* = 296 subjects), stage II (*n* = 40), stage III (*n* = 22), and stage IV (*n* = 4)). Subjects with stage IV lung cancer were also excluded due to the lack of stage IV survivors, as well as the skewed distribution of survivors and nonsurvivors in the two groups. Thus, only subjects with stages I–III were included in our study (Figure 1). Although stage distribution in the NLST varied with the time of diagnosis, no patients were excluded based on this parameter alone.

In order to compare survival time, “days from randomization to first diagnosis of lung cancer” (column name in the NLST master sheet: candx_days) were subtracted from “days from randomization to date last known alive” (column name: fup_days). We then applied an automatic nonstandard matching (NSM) process (MatLab Inc.) to create two uniform survivor and nonsurvivor groups using the following six matching criteria: age (column: age), gender (column: gender), BMI (column: weight/height^2^), smoking history (column: pkyr), and survival time (column: fup_days – candx_days). The matching process normalized the data using the variances of each criterion and included all possible matches to avoid the confounding effects of selection bias. The final matched cohort consisted of 180 subjects (90 survivors, 90 nonsurvivors), each group with 49 subjects with stage I, 19 with stage II, and 22 with stage III cancer. The time to death among nonsurvivors was 894 ± 542 days (range 14–2399 days) and the follow-up period for survivors was 1660 ± 488 days (range 405–2744 days).

### 2.3. Scan Techniques

LDCT scan parameters were based on a range of values agreed upon in the trial (NLST Medical Physics Working Group Meeting, June 2003). These are comprised of 120–140 kVp, 40–80 mAs, and 1.25–2.0 : 1 helical pitch. Since different recruiting sites had different CT scanners, there were variations in scanning and reconstruction parameters although individual sites were required to adhere to strict guidelines to avoid the confounding effects of different scan parameters [14].

### 2.4. LDCT Image Evaluation

**Figure 2:**
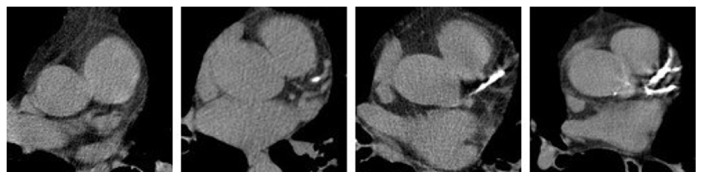
Examples of none, mild, moderate, and severe CAC (0, 1, 2, and 3). The display interval is [40,400].

Two radiologists (AO: 3-year experience; RC: 2-year experience) graded CAC in all 180 CT examinations in a blinded and randomized manner. CAC was graded on a 4-point scale (1 = none, 2 = minimal, 3 = moderate, and 4 = severe) (Figure 2) based on a prior publication on subjective evaluation of CAC on LDCT for lung cancer screening [9]. The radiologists were provided with examples of each CAC grade to use as a reference.

A specific MATLAB program (MathWorks, Natick, MA) was created (R.D.) to semiautomatically quantify CAC into similar four grades of severity. The images were uploaded into the MATLAB program and a circular region of interest (ROI) was drawn to restrict the analysis to the heart region. The images were then normalized via the background values. A threshold pixel value (130 HU) was applied to detect the number of pixels above the threshold, which correlated with the CAC score assigned by the program. The exact scores were assigned heuristically based on the distribution of each score given by the radiologists. Because the grade is given based on the number of pixels above the threshold, it is reproducible regardless of the scanner type or vendor. The CAC scores assigned by the program were compared to the radiologist scores but were not included in the analysis.

Severity of emphysema was quantified with online Airway Inspector software (Brigham and Women's Hospital, Boston MA) [10]. All 180 CT examinations were uploaded to the software to obtain the areas of low attenuation (LAA) proportion. The LAA were defined as those with attenuation values of less than −950 Hounsfield units (HU). The software expressed the LAA as a proportion of the total volume of the lungs.

**Figure 3:**
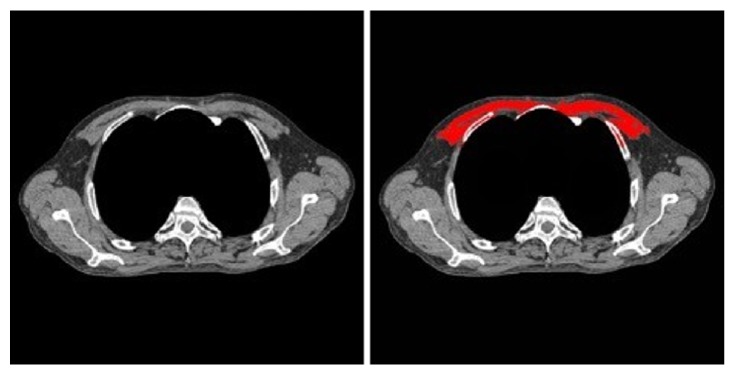
The process used to measure skeletal muscle area (pectoralis major). The display interval is [40,400].

**Figure 4:**
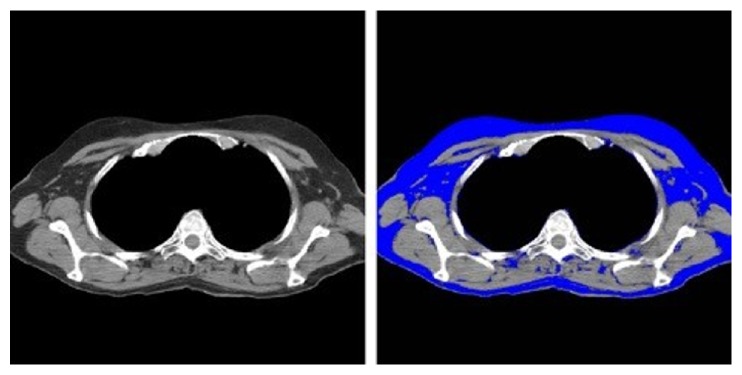
The process used to measure fat attenuation. The display interval is [40,400].

Using a previously described attenuation threshold-based method [10], we used the MATLAB program to segment the pectoralis major muscle at the level of the third thoracic vertebra (T3) (Figure 3). The program also required user input to segment out the mediastinum using a mouse click. The area of the assessed muscle region was measured in CT examinations of all 180 subjects. Since the screening LDCT examinations do not extend up to the midabdomen, we segmented the fat at the level of T3 thoracic vertebra using previously described attenuation threshold method (Figure 4) [11, 12]. The mean fat attenuation was estimated from the mean value of pixels within the accepted thresholds for fat attenuation values (−60 to −120 HU).

### 2.5. Statistical Analysis

Data were analyzed using Microsoft EXCEL, MATLAB software, and SPSS (version 24.0 Armonk, NY: IBM Corp.). The data were stratified according to lung cancer stage, patient smoking history, and history of chronic obstructive pulmonary disease (COPD). A multivariate binary logistic regression model was used to evaluate effects of skeletal muscle area, emphysema, fat attenuation, CAC, tumor stage, age, gender, and height on the likelihood of a subject to be classified as a nonsurvivor over the duration of the NLST. These variables were also included in a multivariate analysis with Cox proportional hazard model. Statistical significance was defined as *p* value less than 0.05.

## 3. Results

There was no significant difference between survivors and nonsurvivors in terms of their age, gender, smoking history, and stage of cancer (*p* > 0.33). Demographic, histology, and staging information for survivors and nonsurvivors is summarized in Table 1. There was no significant difference between histology of detected lung cancers in the surviving and nonsurviving subjects (*p* = 0.18).

Skeletal muscle area (32.6 ± 7.9 cm^2^, 29.2 ± 6.4 cm^2^), fat attenuation (−92.5 ± 4.4 HU, −89.9 ± 4.6 HU), CAC (1.0 ± 1.1, 2.0 ± 1.1), and emphysema (0.13 ± 0.12, 0.15 ± 0.14) in survivors and nonsurvivors were determined with descriptive statistics. The estimated odds ratios for multivariate binary logistic regression model (*χ*^2^(10) = 57.01; *p* < 0.001) are summarized in Table 2. The estimated hazard ratios (HR) and 95% confidence interval (CI) for the Cox regression model (*χ*^2^(10) = 41.52; *p* < 0.001) are summarized in Table 3.

A significant difference was found for CAC among the survivors and nonsurvivors (*p* = 0.004). More coronary artery calcification was found in nonsurvivors (2 ± 1.04) compared to survivors (1 ± 1.08). Although quantitative emphysema between survivors and nonsurvivors was different, however, it did not reach statistical significance (*p* = 0.09).

There was a significant statistical difference in skeletal muscle area among the survivors and nonsurvivors (*p* = 0.0008). Muscle area in the nonsurvivors (29.2 ± 6.4 cm^2^) was significantly lower than in the survivors (32.6 ± 7.9 cm^2^). As opposed to male nonsurvivor versus survivors (31.2 ± 6.2 cm^2^ versus 33.1 ± 7.2 cm^2^, *p* = 0.07), female nonsurvivors had much lower skeletal muscle area compared to female survivors (25.6 ± 4.9 cm^2^ versus 31.7 ± 8.9 cm^2^, *p* = 0.0004). On the other hand, mean attenuation of the muscle showed no significant differences between survivors (57.4 ± 4.4 HU) and nonsurvivors (57.0 ± 4.6 HU) (*p* = 0.38).

Stratified analysis of muscle area with lung cancer stages revealed significant differences for stages 1 and 3 (stage 1: survivors versus nonsurvivors: 32.5 ± 8.9 cm^2^ versus 27.7 ± 6.3 cm^2^, *p* = 0.001; stage 3: survivors versus nonsurvivors: 33.5 ± 7.5 cm^2^ versus 29.8 ± 5.7 cm^2^, *p* = 0.039) but not for stage 2 (stage 2: survivors versus nonsurvivors: 31.6 ± 5.7 cm^2^ versus 31.9 ± 6.4 cm^2^, *p* = 0.44). The difference in muscle area among survivors and nonsurvivors persisted upon stratification based on smoking history (<50 pack-years: survivors versus nonsurvivors: 34.0 ± 7.8 cm^2^ versus 29.9 ± 8.0 cm^2^, *p* = 0.028; and >50 pack-years: survivors versus nonsurvivors: 32.0 ± 7.9 cm^2^ versus 28.7 ± 5.4 cm^2^, *p* = 0.005). Likewise, subjects without history of COPD (31.2 ± 7.5 cm^2^) had much greater muscle area as compared to subjects with COPD (27.4 ± 5.2 cm^2^) (*p* < 0.0001). Muscle area was also substantially lower for nonsurvivors both with and without COPD as compared to survivors (*p* = 0.006).

There was a significant correlation between assessment of CAC by the MATLAB program and the radiologists (*r*^2^ = 0.83).

## 4. Discussion

We found substantial differences in severity of CAC, skeletal muscle area, and attenuation of subcutaneous fat in surviving and nonsurviving NLST subjects in whom lung cancer (stages 1–3) was detected on screening LDCT. Moderate and severe CAC were found to be stronger predictors of death in the assessed subjects than fat attenuation or muscle area. Additionally, a strong difference in severity of emphysema was found between survivors and nonsurvivors. Emphysema has also been reported to increase risk of lung cancer although relationship between severity of emphysema and the risk of lung cancer has not been found to be consistent [15–18]. Emphysema had the highest HR (7.55, 95% CI 1.49–38.29) in our study, which is consistent with the prior cancer risk prediction model of NLST data that reported emphysema as the greatest risk factor of death [19]. Separately in the Multi-Ethnic Study of Atherosclerosis, Oelsner et al. documented strong association between estimated emphysema on chest CT and death from lung cancer death (HR 1.84) [20]. Emphysema is easy to assess and report on LDCT for lung cancer screening but to our best knowledge interpretation of these screening exams do not quantify emphysema on a routine basis.

Our study also documents that moderate (HR 2.91, 95% CI 1.56–5.43) and severe CAC (HR 2.39, 95% CI 1.26–4.53) are independent risk factors of nonsurvival in subjects with screen-detected lung cancers. Jacobs et al. have also reported that CAC on screening LDCT is an independent cardiovascular risk factor for all-cause mortality [21]. Presence of CAC is often commented upon in radiology reports of LDCT screening for lung cancer but in our experience few radiologists attempt to subjectively quantify its severity. Higher hazard and odd ratios for moderate CAC than for patients with severe CAC in our study may be attributed to uneven distribution of CAC among the survivors (*n* = 28 subjects with moderate and severe CAC) and nonsurvivors (*n* = 46 subjects with moderate and severe CAC). Regardless, there were significant differences between CAC among survivors and nonsurvivors (*p* ≤ 0.007) (Tables 2 and 3).

There was a significant increase in subcutaneous fat attenuation among nonsurvivors compared to survivors in our study (HR 1.11, 95% CI 1.06–1.16, *p* < 0.001). This variable has not been assessed in LDCT for lung cancer screening although several publications have established a link between excess deposition of visceral and subcutaneous adipose tissues and excess mortality [22–24]. In addition, this excess fat deposition is associated with adverse levels of adipokines such as adiponectin, leptin, and receptors for leptin, fatty acid, and retinol binding proteins [22–24]. Murphy et al. have also reported a similar association between fat attenuation and adverse levels of adipokines [25]. Increased fat attenuation also correlates with higher adiponectin and lower leptin levels and has been proposed as a biomarker for predicting higher mortality among elderly patients. It is speculated that increased fat attenuation is related to increased collagen but no correlation with inflammatory markers has been reported [25]. Fat attenuation might be a good biomarker for predicting mortality and might be independent of visceral and subcutaneous fat deposition, a finding supported in our study by the BMI of included patients (mean BMI < 30 kg/m^2^).

Although several prior studies have linked sarcopenia or decreased skeletal muscle mass with adverse survival in cancer patients, its use in screening detected lung cancer has not been reported to our best knowledge [26, 27]. Skeletal muscle mass is linearly related to muscle area (used in our study) and is calculated using a regression equation [27]. We found significantly lower muscle area in nonsurvivors as compared to survivors (*p* < 0.001) although HR was just below 1 (95% CI 0.93–0.98) suggesting a weak negative association between muscle area and nonsurvival in subjects with lung cancer detected on screening LDCT.

Prior studies have reported that 20% of COPD patients develop sarcopenia which is associated with increased morbidity and mortality in hospitalized patients; subjects without COPD had higher muscle mass in our study [28]. In our study, the low muscle mass adversely affected subject survival irrespective of clinically diagnosed COPD, a finding that supports the argument low muscle mass is an independent risk factor. In addition, we also noted that female nonsurvivors had significantly lower muscle (*p* = 0.006) than their male counterparts; consistent with prior publications in which women with low skeletal muscle mass had more dose-limiting drug toxicity, when undergoing chemotherapy for stage II/III colon cancer [29].

The main implication of our study pertains to the importance of stating emphysema and CAC in reports of lung cancer screening LDCT. Due to their prognostic significance, radiologists should quantify these findings either subjectively or objectively and state these details in the reports. On the other hand, statistically significant but weak association of lung cancer mortality with fat attenuation and muscle area implies that status quo may be continued at least until automated tools for quantifying these variables are available. Alternatively, a prospective study with larger sample size may be necessary to further question the association between fat attenuation and skeletal muscle area. Semiautomatic assessment of emphysema, CAC, skeletal muscle mass, and fat attenuation in our study have implications as well. Increasingly, that deep learning based software is now being applied to detect and quantify these findings and others (such as fatty liver and bone density) in an automated, objective manner. Although our study did not directly assess these deep learning based algorithms, they provide an insight into their potential uses in patients undergoing LDCT for lung cancer screening.

The US Preventive Services Task Force (USPSTF) recommends LDCT for lung cancer screening for eligible subjects based on their smoking history, age, and ability or willingness of having lung cancer curative surgery [30]. Since results of NLST in generally healthy subjects do not apply to subjects with substantive comorbidities lung cancer screening with LDCT must be discontinued in subjects with considerably reduced life expectancy or inability or unwillingness to undergo surgical cure of detected lung cancer. In this context, our study highlights need for further clinical trials to understand if presence and severity of imaging based variables such as those assessed in our study should be considered when making decision regarding further screening examinations after the initial baseline LDCT screening. Such studies can also help determine if assessment categories for lung cancer screening LDCT with Lung-RADS™ (American College of Radiology) should be modified to consider imaging based signs of potentially serious comorbidities which may be clinically silent.

Our study has limitations. Matching of subjects for age, gender, smoking history, and the stage of lung cancer substantially decreased the actual study size which may have introduced unintentional bias. Conversely, without careful matching, several confounding factors could have skewed the results. In addition, the skeletal muscle mass and mean fat attenuation were determined using a single image. Since image selection affects the measurements, volume measurement is ideal from an accuracy perspective. Though volume measurements could have delivered different results than those presented above, we did not perform such measurements due to software, computational, and time constraints. Another limitation pertains to its retrospective nature which limited the availability of data and may have constrained the full extent of the research. This made it more difficult to ensure that outcomes were measured consistently or using the same criteria. Another limitation was the reliance on the NLST data for lung cancer screening LDCT obtained from multiple different CT scanners using varying scanning and reconstruction parameters. Such variations in scanners, scan parameters, and reconstruction settings can affect the reproducibility of the quantitative results in our study. Although we employed scanner, scan and reconstruction parameters agnostic software for quantifying the assessed variables, accuracy, and predictably of these variables might be dissimilar under more controlled circumstances. Likewise, other limitations of NLST including the shorter follow-up duration of subjects found to have lung cancer also apply to our study as well. A limitation of NLST that extends to our study pertains to lack of data regarding specific genetic mutations for the detected lung cancer, which could be a confounding factor.

In conclusion, emphysema and coronary artery calcification are substantive predictors of survival of lung cancer detected on lung cancer screening LDCT. Increased fat attenuation and low skeletal muscle area are weak but significant predictors of lung cancer mortality in the screening population. While reporting LDCT examinations, radiologists should comment on severity of emphysema and coronary artery calcifications.

## Figures and Tables

**Table 1:** Characteristics of the subjects in the survivor and nonsurvivor groups (*p* > 0.05 for all characteristics).

Characteristics	Survivors	Nonsurvivors
Age	64 ± 5 years	64 ± 6 years
Sex ratio (males : females)	54 : 36	56 : 34
Height (in m)	1.7 ± 0.1	1.7 ± 0.1
Weight (in Kg)	80 ± 16	81 ± 18
BMI (Kg/m^2^)	26.5 ± 4.3	27.0 ± 4.8
Histology (*n*)		
Adenocarcinoma	52	45
Squamous cell cancer	24	26
Small cell cancer	6	4
Large cell cancer	0	5
Unspecified cancers	8	10
Follow-up period in days (range)	1660 ± 488 (405–2744)	894 ± 542 (14–2399)

**Table 2:** Multivariate binary logistic regression analysis demonstrated that emphysema and moderate to severe CAC were the strongest predictors for classification of a subject as a nonsurvivor over the NLST duration. Numbers in parenthesis represent 95% confidence interval.

Variables	Odds ratio (OR)	Coefficient	*p* value
Muscle area	0.89 (0.84–0.94)	−0.12	<0.001
Fat attenuation	1.26 (1.14–1.39)	0.23	<0.001
Emphysema	33.78 (1.99–572.17)	3.52	0.015
Minimal CAC	1.84 (0.72–4.69)	0.61	0.201
Moderate CAC	6.30 (2.29–17.32)	1.84	<0.001
Severe CAC	4.28 (1.54–11.90)	1.45	0.005

**Table 3:** Cox proportion hazard model suggested that emphysema and moderate to severe CAC were stronger predictors of nonsurvival in patients with lung cancer detected in the NLST compared to skeletal muscle area and fat attenuation. Numbers in parenthesis represent 95% confidence interval.

Variables	Hazard ratio (HR)	Coefficient	*p* value
Muscle area	0.96 (0.93–0.98)	−0.04	0.008
Fat attenuation	1.11 (1.06–1.16)	0.10	<0.001
Emphysema	7.55 (1.49–38.29)	2.02	0.015
Minimal CAC	1.20 (0.62–2.33)	0.18	0.588
Moderate CAC	2.91 (1.56–5.43)	1.07	0.001
Severe CAC	2.39 (1.26–4.53)	0.87	0.007

## Data Availability

The authors will be happy to share their measurements with interested investigators or readers. Access to NLST image data and coded subject data require permission from the National Institute of Health.
